# Integrating the MARTINI2 coarse‐grained force field into HADDOCK3 for faster modeling of large biomolecular complexes

**DOI:** 10.1002/pro.70793

**Published:** 2026-09-21

**Authors:** Raphaelle Versini, Victor Reys, Anna Kravchenko, Rodrigo V. Honorato, Alexandre M. J. J. Bonvin

**Affiliations:** ^1^ Bijvoet Centre for Biomolecular Research, Faculty of Science—Chemistry Utrecht University Utrecht The Netherlands

**Keywords:** coarse‐graining, docking, integrative modeling, protein‐nucleic acid complexes, protein–protein complexes

## Abstract

The integration of coarse‐grained (CG) approaches into docking workflows offers a powerful strategy for modeling large biomolecular assemblies with reduced computational costs. We present here the implementation of the MARTINI2 CG force field into the HADDOCK3 integrative modeling platform. This development enables the use of the CG representations and parameters within HADDOCK3 for efficient sampling and scoring of large macromolecular complexes, including protein–protein and protein‐nucleic acid complexes. The implementation takes advantage of the modular and flexible architecture of HADDOCK3, allowing a seamless combination of MARTINI2 representation with the various modules. Conversion from and to all‐atom models is integrated into the CG modeling workflow. The performance of the protocol is first assessed on protein–protein and protein‐DNA benchmarks and then illustrated on a few representative large‐scale systems, demonstrating a significant reduction in computational costs while maintaining biologically relevant accuracy. HADDOCK3 is freely available from https://github.com/haddocking/haddock3.

## 1 INTRODUCTION

Biomolecular interactions govern all essential processes within a living organism, with proteins being key components of those molecular processes. Structural characterization of these interactions remains a challenge, as their often transient nature and other experimental factors can limit the acquisition of reliable structural information (Nooren & Thornton, 2003; Wass et al., 2011).

Computational modeling of protein structures and protein–protein interactions has made a lot of progress in the last 10 years. In fact, artificial intelligence (AI) is now able to predict protein–protein interactions quite accurately (Baek et al., 2021; Evans et al., 2021; Jumper et al., 2021; Lin et al., 2023; ByteDance AML AI4Science Team, 2025), as well as interactions involving nucleic acids, lipids, small molecules and post‐translational modifications (Abramson et al., 2024; Baek et al., 2024; Krishna et al., 2024). However, classical physics‐based docking approaches, such as HADDOCK (Giulini et al., 2025; Honorato et al., 2024), remain relevant and complementary to machine learning architectures, as exemplified by antibody–antigen complexes, which can be reliably modeled by a combination of AI and physics‐based docking methods using interaction restraints to drive the docking (Ambrosetti et al., 2023; Giulini et al., 2024; Xu et al., 2025), or protein‐glycan complexes (Ranaudo et al., 2024) for which current AI models lack support.

Although docking is efficient for small‐ and medium‐sized proteins, its application to large biological systems faces substantial computational demands to sample complex conformational landscapes, while AI‐based approaches are limited by the maximum number of residues they can effectively handle. Coarse‐grained (CG) models can mitigate the limitations of physics‐based methods by associating several heavy atoms into one larger particle or bead (Rader, 2010; Saunders & Voth, 2012; Takada, 2012), which effectively reduces the number of particles to take into account in the simulations. Several docking programs use CG representations to reduce computational costs, enabling efficient exploration of large conformational spaces, and smoothing the interaction energy landscape. For instance, ATTRACT uses reduced protein models to perform systematic docking minimization across thousand initial structures within a reasonable computing time (Vries & Zacharias, 2013). Two other examples using a CG representation are CABS‐doc, a multiscale modeling pipeline (Kurcinski et al., 2015) and OPEP which has been used for rescoring of rigid body protein–protein predictions (Kynast et al., 2016). The MARTINI CG model is currently among the most widely used frameworks for CG simulations; it was first developed for lipids (Marrink et al., 2007) and later extended to include proteins (Monticelli et al., 2008) and DNA (Uusitalo et al., 2015). The MARTINI mapping philosophy is to create one CG particle or bead out of four heavy atoms. This mapping is adapted when atoms are involved in long side chains and cycles, as well as DNA bases. It can represent a wide range of molecular types (high transferability) and enables straightforward back‐mapping to atomistic resolution, making it particularly well‐suited for use in HADDOCK for integrative modeling applications.

The MARTINI2.2 version was previously implemented in HADDOCK2.4 for both proteins (Roel‐Touris et al., 2019) and DNA (Honorato et al., 2019). The main docking steps—rigid‐body docking and semi‐flexible refinement—were performed at the CG level, followed by back‐mapping to all atoms (AA) resolution prior to the final energy minimization. The implementation maintained the overall docking performance, while reducing the computational costs, allowing for the modeling of larger systems and/or faster runs.

When redesigning HADDOCK into a modular version (Giulini et al., 2025), moving away from the rigid workflow with pre‐defined and parametrizable steps into a modular platform enabling the definition of custom workflows, its coarse‐graining feature was deprioritized considering the large scope of the redesign. Here, we describe the implementation of coarse‐graining with the MARTINI2.2 (Monticelli et al., 2008) force‐field into the latest version of our docking software, HADDOCK3 (Giulini et al., 2025). We added two new modules *topocg* and *cgtoaa* that take care of the atomistic to CG mapping and vice versa, a task that was only automated in the web implementation of HADDOCK2.4 (Honorato et al., 2024), while the command‐line version required providing both atomistic and CG models as input. We show on protein–protein and protein‐nucleic acid benchmark datasets that HADDOCK3 CG can maintain the success rate obtained with all‐atom (AA) models while reducing the simulation time. We further illustrate HADDOCK3's CG usage with three different cases: (1) the human liver phosphofructokinase‐1 filament in the R‐state conformation (Lynch et al., 2024), (2) the heptameric KaiC:KaiB 1:6 complex (Ishiura et al., 1998; O'Neill & Reddy, 2011), and (3) the PRC1‐nucleosome core particle complex (McGinty et al., 2014).

## 2 MATERIALS AND METHODS

### 2.1 Integration of the MARTINI2 CG force field into HADDOCK3

The implementation of MARTINI2.2 (Monticelli et al., 2008; Uusitalo et al., 2015) into HADDOCK3 (Giulini et al., 2025) builds upon the previous implementation in HADDOCK2.4 (Honorato et al., 2019; Roel‐Touris et al., 2019). The converted MARTINI topology and parameter files compatible with the HADDOCK engine CNS (Crystallography and NMR System) (Brünger et al., 1998), built for HADDOCK2.4 (Honorato et al.,  2024) were used for this work, including the MARTINI2 (Uusitalo et al., 2015) extension to DNA.

Two new modules were created to enable CG docking. The *topocg* module handles the mapping of the AA input structures into their CG representation and prepares the corresponding CG topology files. This module can only be used after the *topoaa* module, which creates the AA topologies and builds any missing atoms. The refinement module *cgtoaa* performs the reverse operation, that is, the back‐mapping of the CG structures into their AA representation using distance restraints (see below).

The MARTINI (Monticelli et al., 2008; Uusitalo et al., 2015) mapping philosophy was kept in the *topocg* module, following a four‐to‐one (4:1) rule for the amino acids' backbone, meaning all four heavy atoms (N, C_α_, C, O) are represented by a single particle (bead) placed at their center of mass. It was derived from the original Martinize script (Monticelli et al., 2008). The conversion rule is adapted for side chains, and ranges from 4:1 to 2:1 mapping, as well as “small” beads in rings (HIS, PHE, TYR, TRP). As the backbone bead parameters depend on the secondary structure of the molecule, we are using DSSP to determine and encode the secondary structure into the B‐factor field of the AA models. The proper parameters are then automatically selected for each backbone bead. If no secondary structure is found, random coil parameters are applied instead (which is also the behavior in case DSSP is not installed). Note that the definition of the secondary structure in CG representation is not a requirement to maintain it in HADDOCK, as most of the structure is kept rigid during the semi‐flexible refinement stage. The backbone of DNA is represented by 3 beads, one for the phosphate group (4:1) and two for the sugar (3:1). Bases are mapped into three and four beads for pyrimidines and purines, respectively. The MARTINI2 force‐field considers four types of interactions for proteins (Monticelli et al., 2008): polar (P), nonpolar (N), apolar (C), and charged (Q). For nucleic acids, eight beads are added to mimic hydrogen bonding formed between complementary nucleotide base pairs (Uusitalo et al., 2015), contributing to the stabilization of the DNA double helix structure.

The back‐mapping procedure (*cgtoaa* module) makes use of HADDOCK's versability in using distance restraints. During the initial CG mapping stage, atoms‐to‐bead restraints are defined, consisting of one distance restraint per bead of 0 Å (lower and upper distance defined as 0) between the geometric center of the atoms and the bead to which they belong. These restraints are used to morph the starting respective AA single structures onto the CG modeled complex. This back‐mapping protocol consists of three main steps: (i) An initial fitting of the single AA structures onto their CG counterparts using the atoms‐to‐bead distance restraints, downscaling the intermolecular interactions to avoid clashes between molecules; (ii) short cycles of restrained minimization and molecular dynamics introducing conformational changes into the AA structures to match the conformation of the CG model, gradually increasing the interaction strength between molecules; and (iii) final clash removal through additional rounds of minimization and brief molecular dynamics (MD) simulations. During all those steps, the CG model is kept rigid and fixed in space, and both AA and CG models co‐exist, but do not see each other except through the effect of the CG to AA distance restraints. At the end of the protocol, the CG model is deleted. For more information, refer to the Methods section of Roel‐Touris et al. (2019).

### 2.2 Docking protocols

All docking calculations were performed locally using HADDOCK3 (Giulini et al., 2025). For comparison purposes, two docking workflows were performed, one with the standard AA representation using the united‐atom OPLS force field (Jorgensen & Tirado‐Rives, 1988) and one in which docking and semi‐flexible refinement are performed at the CG level with MARTINI2.2 (Monticelli et al., 2008), preceded by an AA to CG conversion module (*topocg*) and followed by the back‐mapping module (*cgtoaa*).

**FIGURE 1:**
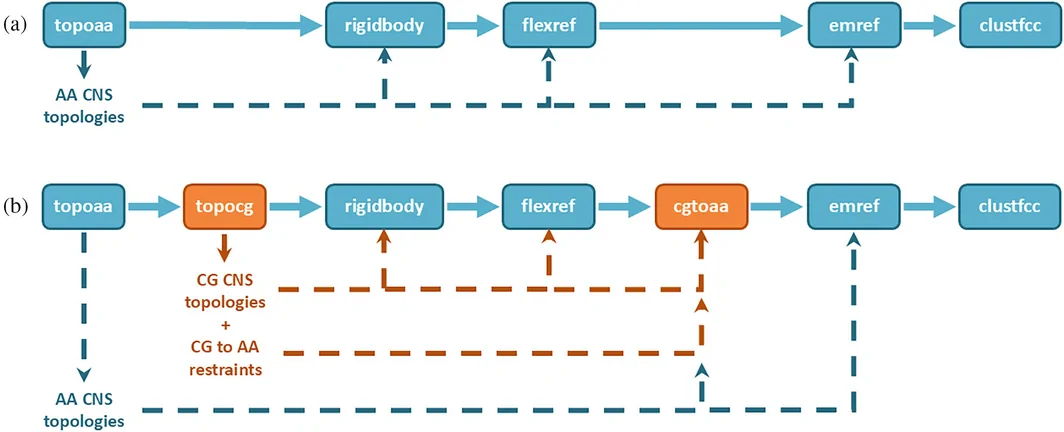
All‐atom (AA) (panel a) and coarse‐grained (CG) (panel b) HADDOCK3 docking workflows. The two orange steps are only present in the CG workflow. *topoaa* defines the AA topology of the system and builds any missing atoms. *topocg* then maps the AA structures into their CG counterparts and defines the CG topology of the system. The *rigidbody* module performs the rigid body docking using respective topologies (AA in AA workflow and CG in CG workflow). *seletop* selects the top X models (X = 200 in our benchmarks). The *flexref* module performs the semi‐flexible interface refinement step, the *cgtoaa* module performs the back‐mapping from CG representation to AA, and *emref*, the final energy minimization (in AA representation in both cases). The *clustfcc* module then performs a fraction of common contact‐based clustering. Dashed lines represent the usage of topology and restraint files created by the two topology modules.

The workflow is illustrated in Figure 1. A HADDOCK3 run always starts with the *topoaa* module, which defines the topology of the system and builds any missing atoms. In the CG pipeline, this is followed by the *topocg* module. Rigid body docking (*rigidbody*) is then performed. The topX models (with X = 200 (default) or 400 in case of increased sampling) are then selected (*seletop*), followed by the *flexref* module, which performs a semi‐flexible interface refinement of the models following a simulated annealing MD protocol in torsion angle space. In the CG pipeline, we then proceed to use the *cgtoaa* module to back‐map the CG structures into their AA counterparts. The two final steps of the docking are the energy minimization (*emref*) module and the fraction of common contact‐based clustering (*clustfcc*) module (with contacts identified with a cutoff distance of 7 Å for the CG pipeline, instead of 5 Å for AA). To analyze the quality and convergence of the models we added *caprieval* modules at various stages during the workflow (see Metrics for Evaluation of Model Quality below).

The HADDOCK3 sampling (rigidbody) and refinment (flexref, emref) modules automatically adapt the non‐bonded parameters to the representation of the model. In CG representation, we use a dielectric constant of 10, with a 14 Å non‐bonded cutoff and a switching function applied between 12 and 14 Å. For AA representations, a short cutoff of 8.5 Å switched at 6.5 Å is used (default in all HADDOCK versions) together with a distance‐dependent dielectric constant (1/*r*). For protein‐DNA docking, a constant dielectric is used (dielec = cdie) set to 78 by adding to the relevant modules (*rigidbody, flexref* and *emref*) of the workflow epsilon = 78 and epsilon_cg = 78.

The HADDOCK3 workflows used for protein–protein and protein‐DNA benchmarking are shown in Supplementary Material Figures SI‐1 and SI‐2 and available from the Git repositories of the benchmarking datasets.

### 2.3 Protein–protein and protein‐DNA docking benchmarks

To systematically test the performance of our CG protocol on protein–protein complexes, the protein–protein Docking Benchmark version 5.0 (Vreven et al., 2015), containing 229 unbound–unbound cases, was used. A HADDOCK‐ready version of this benchmark can be found on GitHub at https://github.com/haddocking/BM5-clean. Default sampling parameters were used (1000/200/200 for *rigidbody*, *flexref* and *emref*, respectively). The ambiguous interaction restraints (AIRs) guiding the docking were defined using the interfaces defined from the bound complex (true interface) by selecting all residues making intermolecular heavy atom contacts within a 5.0 Å cutoff. These restraints model a rather ideal situation in which the interacting residues at the interface are known, even though their exact pairwise contacts are not, as would be the case when using experimental data such as NMR chemical shift perturbations or hydrogen/deuterium exchange mass spectrometry (Karaca & Bonvin, 2013; Rodrigues & Bonvin, 2014). Each active residue on one binding partner is ambiguously restrained to all active residues on the other. This network of highly ambiguous restraints brings the interfaces together without pre‐defining their relative orientations. As a result, the docking process retains substantial conformational and orientational freedom and does not correspond to a fully constrained reconstruction of the native complex.

For protein‐DNA docking, we used 44 unbound‐unbound cases from the protein‐DNA benchmark (Honorato et al., 2019; van Dijk & Bonvin, 2010). A HADDOCK‐ready version of the benchmark can be found on GitHub at https://github.com/haddocking/Prot-DNABenchmark. AIRs guiding the docking were taken from Van Dijk et al (van Dijk & Bonvin, 2010). (available from the Git repository).

Note that, as the main purpose of this work was to re‐implement and compare AA vs. CG docking performance, we did not perform any ab‐initio docking benchmarking.

### 2.4 Scoring

HADDOCK's scoring function is used after each major docking steps (rigid body docking, semi‐flexible refinement and energy minimization) to select and/or rank models. It incorporates the intermolecular electrostatic (*E*_elec_) and van der Waals (*E*_vdW_) energies, an empirical desolvation energy term (*E*_desolv_) (de Vries et al., 2007; Fernández‐Recio et al., 2004), the buried surface area (BSA), and the ambiguous interaction restraint violation energy (*E*_air_). The specific weights and contributions of these terms vary depending on the step of the docking protocol:

(1) $${\text{HADDOCK}}_{\text{rigidbody}}=\begin{array}{l} 0.01\;E_{\mathrm{vdw}}+1.0\;E_{\text{elec}}+\;1.0\;E_{\text{desolv}}\\ +0.01\;E_{\mathrm{air}}–0.01\;\mathrm{BSA} \end{array}$$

(2) $${\text{HADDOCK}}_{\text{flexref}}=\begin{array}{l} 1.0\;E_{\mathrm{vdw}}+1.0\;E_{\text{elec}}+1.0\;E_{\text{desolv}}\\ +\;0.1\;E_{\mathrm{air}}–0.01\;\mathrm{BSA} \end{array}$$

(3) $${\text{HADDOCK}}_{\text{emref}}\begin{array}{l} =1.0\;E_{\mathrm{vdw}}+0.2\;E_{\text{elec}}+1.0\;E_{\text{desolv}}\\ \;+0.1\;E_{\mathrm{air}} \end{array}$$

For the CG pipeline both the rigid‐body docking and flexible refinement are performed at the CG level, where *E*_vdW_ and *E*_elec_ are the energy terms calculated using a 12–6 Lennard‐Jones and Coulomb potential, respectively, with the MARTINI force‐field parameters. Any steps performed in AA use the OPLS force‐field parameters for *E*_vdW_ and *E*_elec_ energy terms.

### 2.5 Metrics for evaluation of model quality

The quality of the generated models was evaluated using the *caprieval* module of HADDOCK3, which calculates the CAPRI metrics (Lensink & Wodak, 2010), fraction of native contacts (FNAT), interface (i‐RMSD) and ligand (l‐RMSD) root‐mean‐square deviations from the reference experimental structure if provided (otherwise it uses the best‐ranked model of each stage of the workflow). FNAT is calculated using all heavy atom–heavy atom intermolecular contacts using a 5 Å distance cutoff (CAPRI definition) (Lensink & Wodak, 2010) for AA structures and a 7 Å cutoff for CG representations, to account for the different resolution (based on contact analysis between the two representations, results not shown). CG reference structures are provided to *caprieval* during the CG stage of the workflow. The i‐RMSD is calculated on the interface backbone after superimposition on the interface residues, defined as those with any heavy atom within a 10 Å distance of the partner molecule. The l‐RMSD is calculated on the ligand (usually the smallest molecule) after superimposition on the backbone atoms of the receptor (largest molecule). For both i‐RMSD and l‐RMSD, only backbone heavy atoms are considered for the proteins (C_*α*_, C, N, O) and base heavy atoms for the DNA. Note that *caprieval* also returns the DockQ score (Basu & Wallner, 2016) (a combination of i‐RMSD, l‐RMSD and FNAT) and the interface ligand RMSD score (il‐RMSD), which is useful for small molecules quality assessment. It is calculated by fitting the interface backbone of the receptor and calculating the RMSD of the ligand.

Based on these metrics, the quality of the docking poses is classified as:

- *High*: FNAT ≥0.5 and i‐RMSD≤1 Å or l‐RMSD≤1 Å (0.8 <DockQ)
- *Medium*: FNAT ≥0.3 and 1 Å< i‐RMSD≤2 or 1 Å l‐RMSD ≤5 Å (0.49< DockQ <0.8)
- *Acceptable*: FNAT ≥0.1 and 2 Å< i‐RMSD≤ 4 or 5 Å< l‐RMSD≤ 10 Å (0.23< DockQ< 0.49),
- *Near‐Acceptable*: FNAT ≥0.1 and 4 Å< i‐RMSD≤ 6 Å,
- *Low quality*: FNAT <0.1 or i‐RMSD >6 Å or l‐RMSD >10 Å.

### 2.6 CG integrative modeling examples with HADDOCK3

- PFK filament

As a first application example, the phosphofructokinase‐1 (PFK) filament in the R‐state conformation was modeled (reference cryo‐EM structure PDB ID: 8W2I (Lynch et al., 2024)). This complex is composed of two R‐state tetramers for a total of 5928 residues. To construct the filament from the unbound R‐state conformation, the homotetramer from the cryo‐EM structure of the R state (PDB ID: 8W2G (Lynch et al., 2024)) was used as a starting point. The AIRs were defined from the interfaces extracted from the reference structure using the *ti* option from *haddock‐restraints* (https://github.com/haddocking/haddock-restraints). Solvent accessible residues within a 3.9 Å cutoff from the partner molecule were selected to define the ambiguous interaction restraints. The list of residues used for the docking is provided in Supplementary Table SI‐1. Default sampling was used: 1000/200/200 for *rigidbody*, *flexref* and *emref*, respectively.

- KaiC‐KaiB complex

To model the KaiC:KaiB 1:6 complex, a single seven‐molecule docking run was performed, targeting the CI domain of KaiC (see Snijder et al., 2014) for details). The crystal structure of KaiC (PDB ID: 3DVL (Pattanayek & Egli, 2009), comprising 12 domains arranged in two six‐membered rings, was used as the starting model. For KaiB, six copies of an NMR structure (PDB ID: 5JYT (Tseng et al., 2017)) were used, which exhibits the same fold as in the reference complex, while the crystal structure (Villarreal et al., 2013) originally used in Snijder et al. (Snijder et al., 2014) corresponds to a fold‐switched conformation. The cryo‐EM structure of the KaiC:KaiB:KaiA complex was used as a reference (PDB ID: 5N8Y (Snijder et al., 2017)).

Residues previously identified by HDX‐MS as solvent‐protected in the CI domain of KaiC, and in KaiB (Snijder et al., 2014), were defined as active and passive residues, respectively, after filtering for solvent accessibility (relative residue solvent accessibility >50% as calculated with NACCESS (Lee & Richards, 1971)). A detailed list of these residues is provided in Supplementary Table SI‐2.

The KaiB monomers were restrained to an approximate C6 symmetry by defining three C2 symmetry pairs (B–E, C–F, D–G) and two C3 symmetry triplets (B–D–F and C–E–G).

In addition to the symmetry restraints, we established custom symmetry restraints which respect the C3 restraints, which ensures again the similarities between the 6 KaiB defined. As symmetry restraints were applied, sampling of 180° rotations during the rigid‐body docking stage was disabled. The random removal of ambiguous restraints was removed as well. Furthermore, because of the number of subunits involved (7 in total), the sampling was increased to 10,000/400/400 for *rigidbody*, *flexref*, and *emref*, respectively.

- PRC1 Ubiquitination module bound to the nucleosome

To model the Polycomb repressive complex 1 (PRC1) Ubiquitination module bound to the Nucleosome, the unbound crystal structure of the enzymatic complex PRC1 was used (PDB code: 3RPG) (Bentley et al., 2011), and docked with the nucleosome particle (PDB code: 3LZ0). The default sampling values were used.

The docking was guided by interaction restraints reported in the literature available at the time of CAPRI Round 31 (October 2014). These included a single unambiguous distance restraint (with an upper limit of 2 Å) between the SG atom of the catalytic cysteine (Cys85) of PRC1 and the NZ atom of Lys118 on chain C of histone H2A, the ubiquitination site. In addition, mutagenesis data identifying key PRC1 residues (K62 of chain B, R64 of chain B, K97 of chain C, and R98 of chain C, original PDB numbering) as essential for nucleosome binding were incorporated (Bentley et al., 2011; Mattiroli et al., 2014). AIRs were defined by assigning these PRC1 residues as active and all solvent‐accessible histone residues on the side of the nucleosome where the catalytic cysteine is found as passive, filtered for solvent accessibility (the restraints and input structures were the same as in the protocol described in the HADDOCK2.4 server paper (Honorato et al., 2024)). A complete list of the active and passive residues used to guide the docking is provided in Supplementary Table SI‐3. Default HADDOCK3 settings were used for this docking run.

## 3 RESULTS AND DISCUSSION

The docking protocol using MARTINI2, first implemented in HADDOCK2.4 (Honorato et al., 2019; Roel‐Touris et al., 2019), was ported to HADDOCK3 (Giulini et al., 2025) through the addition of two modules: the *topocg* module, which maps the AA structures to their CG counterparts, and the *cgtoaa* module, which performs the back‐mapping of the CG structures into their AA representation. In the following section, we first discuss the performance of this implementation, comparing AA and CG docking results for the protein–protein and protein‐DNA benchmarks. We then showcase the HADDOCK3 CG docking protocol by modeling three large complexes.

### 3.1 Overall AA versus CG performance for protein–protein and protein‐DNA docking

**FIGURE 2:**
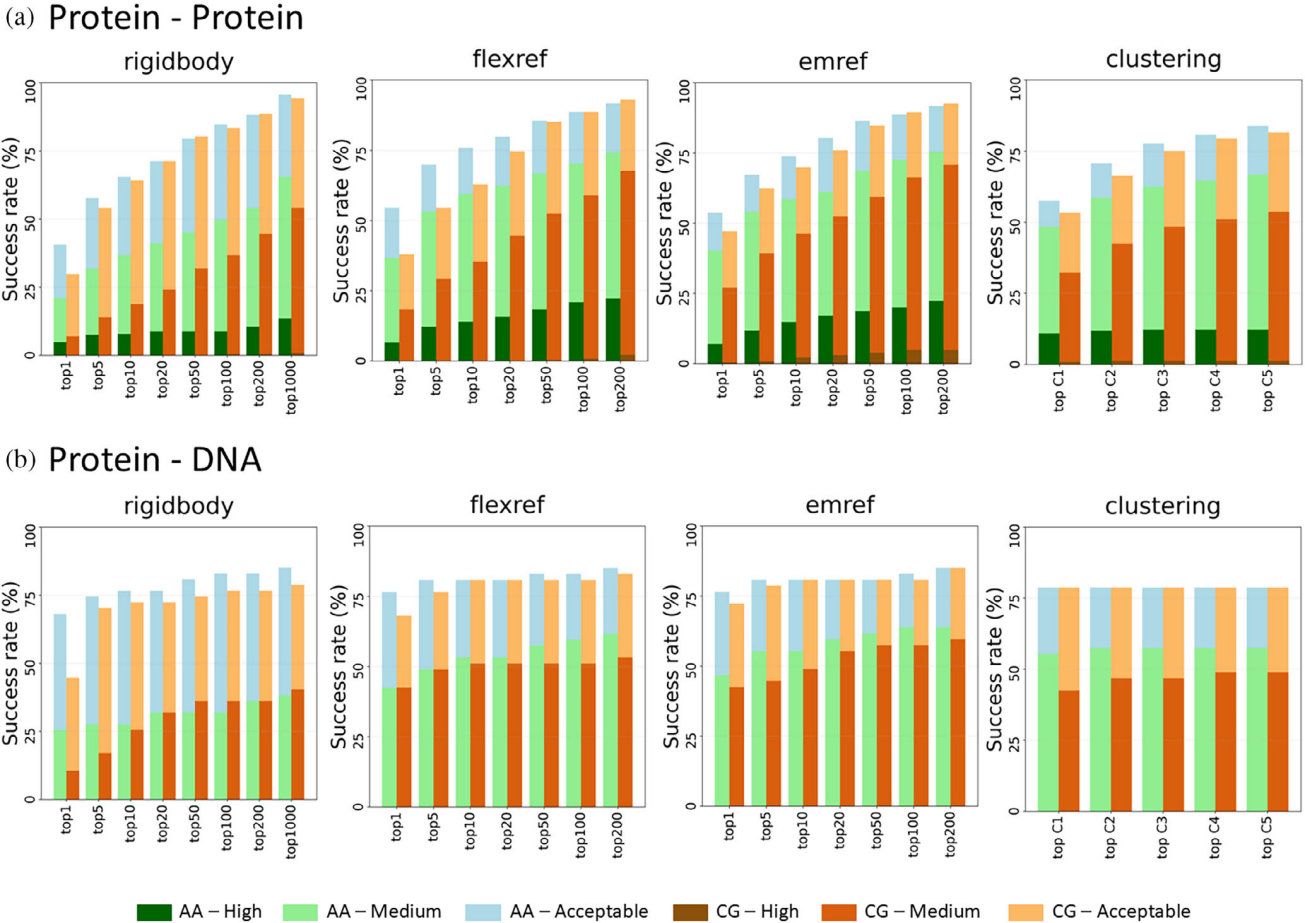
Performances of the AA and CG docking protocols in HADDOCK3. (a) Overall success rate (%) of the protein–protein benchmark as a function of the number of models considered (topX), comparing AA and CG protocols. For the clustering analysis, the top four models from the highest‐ranked clusters were considered, with “top C1” referring to the first‐ranked cluster, “top C2” to the two highest‐ranked clusters, and so on. (b) Same as (a) but for the protein‐DNA benchmark.

Two datasets were used for this comparison: the BM5 protein–protein benchmark (Vreven et al., 2015) (https://github.com/haddocking/BM5-clean), composed of 229 complexes, and the protein‐DNA benchmark (van Dijk & Bonvin, 2008) (https://github.com/haddocking/Prot-DNABenchmark), consisting of 44 complexes. The docking was performed from the unbound structures of each protein and driven by ambiguous interaction restraints derived from the real interface, which imitates an ideal scenario for HADDOCK3. The aim was not to provide a full benchmarking of HADDOCK capabilities under various scenarios, but rather to compare the AA and CG implementations. The success rates at various stages throughout the docking were analyzed as the percentage of cases for which an acceptable or better model was predicted in the Top N models (Figure 2).

The overall docking performance achieved at the AA level for the protein–protein benchmark is largely preserved in the CG approach for each step of the docking. After the final energy minimization step, the top 1 success rate for acceptable or higher quality is 53.7% and 47.2% for AA and CG protocols, respectively (Figure 2a). The success rate in the top 10 reaches 73.8% for AA and 69.9% for CG. In the top 5 clusters, the success rate reaches 83.8% and 81.6% for the AA and CG protocols, respectively. When evaluating model quality, the AA protocol generates higher quality models than the CG protocol (Figure 2a).

Protein‐DNA benchmarking showed similar success rates between the CG and AA pipelines as well, as shown in Figure 1c. Interestingly, after the refinement stage (*emref*), the CG protocol achieved very similar overall performance as the AA approach, with top1 success rates of 72.3% versus 74.5% and top5 success rates of 78.7% versus 80.8%, respectively. This consistency is also observed after clustering, as seen for the top1 ranked cluster, which reaches success rates of 78.7% for both protocols, in line with the previous implementation of CG protein‐DNA docking in HADDOCK2.4.

The success rate for the larger protein–protein complexes is coherent with the previous HADDOCK2.4 CG implementation (Roel‐Touris et al., 2019) as well (results not shown). However, similar to the protein–protein benchmark, the quality of the models is higher for the AA docking as measured by the success rates of medium quality models (Figure 2c). As the DNA was modeled as a perfect B‐form helix, the amount of conformational changes between unbound and bound form prevents the obtention of high‐quality models using a single docking round (a two‐stage protocol leading to higher quality models was previously described (van Dijk & Bonvin, 2010)).

### 3.2 Computational efficiency

**FIGURE 3:**
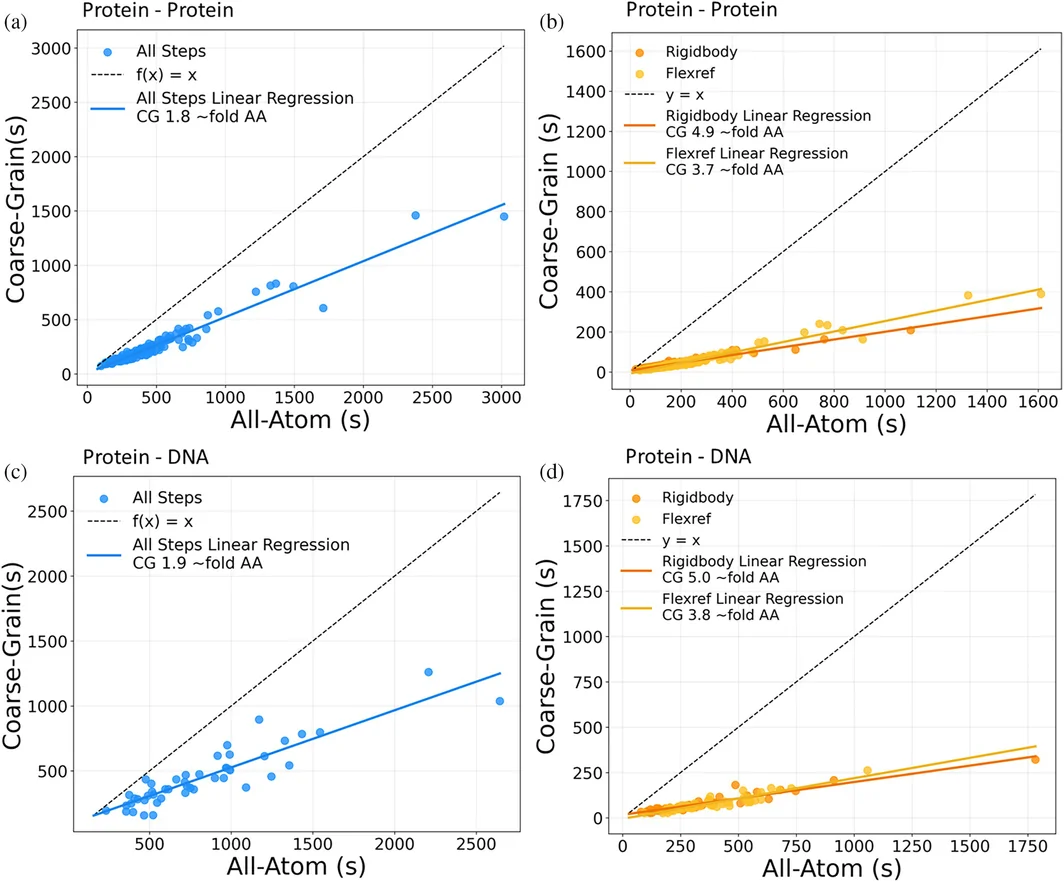
Correlation plot of total run time for CG versus AA simulations. (a) and (b): Run times for the protein–protein benchmark, with all of the steps of the workflow (left), or only the *rigidbody* and *flexref* modules (right). (c) and (d): Run times for the protein‐DNA benchmark.

Coarse‐graining reduces the number of degrees of freedom in the system, thereby improving the computational efficiency of docking calculations. As shown in Figure 3a,c, CG docking runs (full workflows combining pre‐ and post‐processing stages) are two‐fold faster than their AA counterparts for both the protein–protein and protein–DNA benchmarks. A separate analysis of the most computationally expensive steps, *rigidbody* and *flexref*, modules reveals an even larger performance gains, with speedups of 4.9‐ and 3.7‐fold, respectively, for the protein–protein benchmark, and 5.0‐ and 3.8‐fold, respectively, for the protein–DNA benchmark (Figure 3b,d). This difference in speed‐up between specific stages and the overall workflow comes from the pre‐ and post‐processing stages and, in particular, the *cgtoaa* back‐mapping step in the CG protocol, which adds additional computation requirements to the full workflow.

### 3.3 CG integrative modeling examples with HADDOCK3

#### 3.3.1 The PFK filament: Modeling a dimer of large homotetrameric proteins

The practical capabilities of the HADDOCK3‐CG approach were first illustrated using the phosphofructokinase‐1 (PFK) test case. Two R‐state PFK tetramers (PDB ID: 8W2G) were used to construct the filament structure. Interaction restraints were taken directly from the reference structure (PDB ID: 8W2I), mimicking an ideal docking case. The overall docking pipeline is similar to the protein–protein benchmark one, with sampling of 1000/200/200 for the rigid‐body docking, flexible refinement, and energy minimization respectively.

**FIGURE 4:**
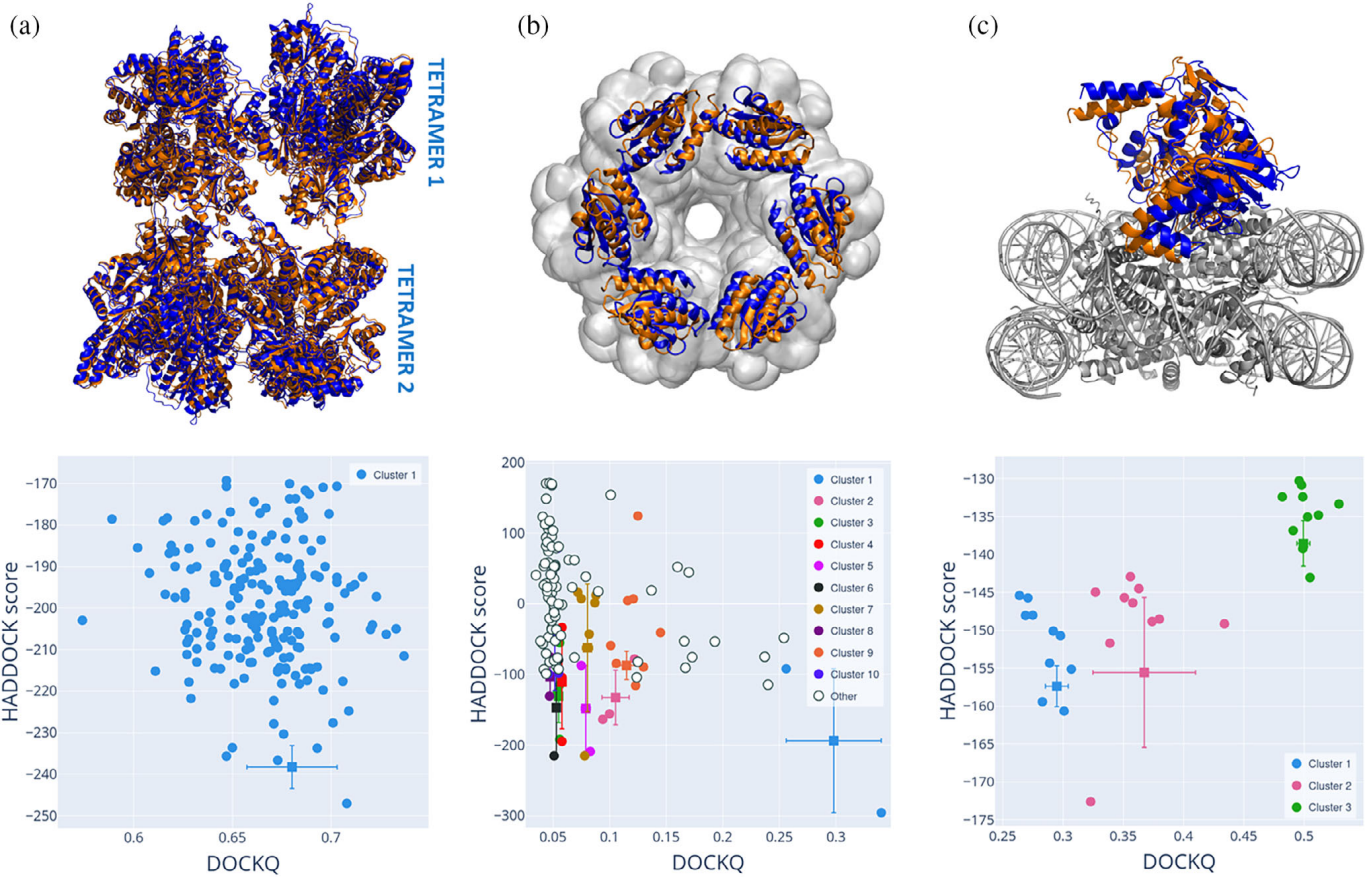
Structure comparison of top‐ranking models predicted by HADDOCK3‐CG. (a) Top1 ranked model of PFK R‐state filament (orange) superimposed with the reference (blue, PDB ID: 8W2L). On the second panel, the DockQ of each model after the clustering phase is shown as a function of the HADDOCK score. (b) Top1 ranked CG model of KAiC‐KaiB (orange) superimposed onto the cryo‐EM model (blue, PDB ID: 5N8Y). On the second panel, the DockQ of each model after the clustering phase is shown as a function of the HADDOCK score. (c) Top1‐ranked (orange) CG model of PRC1 superimposed onto the experimental crystal structure (blue, PDB ID: 4R8P). On the second panel, the DockQ of each model after the clustering phase is shown as a function of the HADDOCK score.

The best‐ranked model after the last energy minimization step was analyzed. The top‐ranked prediction was classified as a medium‐quality model according to CAPRI criteria, exhibiting an i‐RMSD of 1.21 Å, fraction of native contacts of 0.85 and DockQ of 0.71. The model aligned onto the reference structure is shown in Figure 4a. Notably, all models generated at the end of the docking run fall within the medium‐quality category and cluster into a single cluster, highlighting the robustness and consistency of the HADDOCK3‐CG protocol for this test case (using ideal restraints). All cluster statistics are reported in Supplementary Table 4.

#### 3.3.2 KaiC‐KaiB: Symmetry‐restrained docking of a multi‐component protein assembly

The heptameric KaiC:KaiB (1:6) complex was modeled using simultaneous seven‐body docking guided by the same hydrogen–deuterium exchange MS data used in a previous work (Snijder et al., 2014). KaiC consists of two hexamers of CI and CII domains, respectively (Hayashi et al., 2003, 2006). It binds six KaiB monomers (Ishiura et al., 1998). Earlier studies initially suggested two possible binding modes: CI or CII, and the initial modeling pointed to the CII binding mode (Snijder et al., 2014) based on an analysis of MS collision cross‐section data. Subsequent cryo‐EM data (Snijder et al., 2017), however, revealed that KaiB binds to the CI domain in a conformation corresponding to the NMR structure of KaiB (PDB ID: 5JYT) (which differs from the first available crystal structure of KaiB, showing a fold switch (Tseng et al., 2017)). The CI versus CII binding analysis was addressed in the previous implementation of CG in HADDOCK2.4 (Roel‐Touris et al., 2019), demonstrating that docking with the correct KaiB conformation was able to identify the correct binding mode. Here, as an illustration of the HADDOCK3‐CG protocol, we focus exclusively on the CI binding mode, which was modeled using the full KaiC structure and six copies of the binding‐competent KaiB conformation. The same docking procedure was applied as in our previous work (Roel‐Touris et al., 2019), with symmetry restraints approximating C6 symmetry between the six KaiB subunits by defining combined C3 and C2 relationships. In addition to the symmetry restraints, we established custom symmetry restraints, consisting of pairs of symmetrical distances between the various KaiB units and KaiC, which ensure similar binding modes between the 6 KaiB defined.

Analysis of the clustering results revealed that the near‐native solutions were primarily found in the first‐ranked cluster. In fact, the first‐ranked structure (Figure 4b) was classified as an acceptable‐quality model according to CAPRI criteria with an i‐RMSD of 5.0 Å, a FNAT of 0.37, l‐RMSD of 7.30 Å, and DockQ of 0.34. The corresponding metrics for all clusters are reported in Table SI‐5.

#### 3.3.3 CG modeling of the PRC1 Ubiquitination Module in Complex with the Nucleosome

The Polycomb repressive complex 1 (PRC1), which plays a central role in transcriptional repression during development by catalyzing histone ubiquitination (Mattiroli et al., 2014), was used here as our third example to illustrate CG docking. Using interaction restraints derived from the literature (see Materials and Methods), the PRC1–nucleosome core particle complex was modeled with HADDOCK3‐CG and the resulting models were evaluated against the available crystal structure of the complex (PDB ID: 4RP8 (McGinty et al., 2014)).

Based on the HADDOCK score, the top‐ranked model (Figure 4c) from the first cluster is of acceptable quality with a DockQ of 0.32, FNAT of 0.29, l‐RMSD of 7.8 Å, and i‐RMSD of 5.5 Å. The best model of medium quality and with a DockQ of 0.529 was identified in the third‐ranked cluster (6th rank). The top three clusters are, however, all of acceptable quality or higher. Cluster statistics are shown in Supplementary Table 6, showing the first 3 clusters to be in the acceptable to medium quality range. After energy minimization, 196 structures out of 200 are of acceptable or higher quality (27 medium quality). These results indicate that the CG docking protocol can successfully identify near‐native PRC1–nucleosome configurations using literature‐derived interaction restraints.

## 4 CONCLUSIONS

In this work, we implemented the CG MARTINI force‐field into HADDOCK3 and evaluated its performance against AA docking, aiming at extending integrative docking with HADDOCK3 to large and complex biomolecular systems. Benchmarking on protein–protein and protein–DNA datasets demonstrates that the CG protocol largely preserves the docking success rates observed with AA calculations. The reduced representation accelerates simulations by decreasing the number of interactions, enabling faster sampling and making the docking of large assemblies more computationally feasible. The applicability of the CG protocol in HADDOCK3 was further illustrated with three example cases—a phosphofructokinase‐1 filament, the heptameric KaiC:KaiB (1:6) complex, and the PRC1–nucleosome core particle complex—where near‐native models were successfully generated using experimentally derived interaction restraints. Together, these results highlight the usage of CG representation of molecules in HADDOCK3 as a robust and efficient framework for integrative modeling of large biomolecular assemblies.

## AUTHOR CONTRIBUTIONS

**Victor Reys:** Software; writing – review and editing; validation; methodology. **Alexandre M. J. J. Bonvin:** Conceptualization; funding acquisition; methodology; validation; writing – review and editing; software; supervision; resources; investigation. **Raphaelle Versini:** Investigation; writing – original draft; methodology; validation; visualization; formal analysis; software. **Rodrigo V. Honorato:** Software; writing – review and editing; validation; methodology. **Anna Kravchenko:** Validation; writing – review and editing; software; methodology.

## ACKNOWLEGMENTS

The authors acknowledge funding from the European High Performance Computing Joint Undertaking project BioExcel (Center of Excellence for Computational Biomolecular Research) (101093290).

## Supporting information


**Figure SI‐1:** Detailed workflow for the protein–protein benchmark for both AA (A) and CG protocols (B).
**Figure SI‐2**: Detailed workflow for the protein‐DNA benchmark for both AA (A) and CG protocols (B).
**Table SI‐1**: Detailed list of residues used as interaction restraints in the docking process for the PFK filament. Note that the numbering here is that of the experimental structure (PDB ID: 8W2I). For docking the numbering was adapted to that of the pre‐processed input structures with the monomers merged into a single chain with non overlapping numbering.
**Table SI‐2**: Detailed list of residues used as interaction restraints in the docking process for the KaiC‐KaiB complex. Note that the numbering here is that of the experimental structures. As KaiC is an hexamer, the numbering of the six copies was shifted by 1000, 2000, 3000, 4000, 5000, and 6000, respectively, and restraints were defined for each copy, targeting each a specific KaiB monomer. For KaiB, the residues identified from H/D data and their surface neighbors were both treated as passive residues in HADDOCK.
**Table SI‐3**: Detailed list of residues used as interaction restraints in the docking process. Residue numbering for the nucleosome follows a HADDOCK‐adapted scheme, in which residue indices were offset by 200 for each chain (chains 1–10) to ensure unique numbering for each residue.
**Table SI‐4**: Cluster‐based statistics for the PFK filament. HADDOCK score single terms are averaged over the top 4 members of each cluster. Clusters are ordered according to the averaged HADDOCK score (a.u.). Evdw: Lennard‐Jones potential. Eelec: Coulomb potential. EAIR: Ambiguous interaction restraints energy. Edesolv: Empirical desolvation score. BSA: Buried surface area. The CAPRI docking quality metrics are as follows: interface RMSD (i‐RMSD), fraction of native contacts (FNAT), ligand RMSD (l‐RMSD) and DOCKQ.
**Table SI‐5**: Cluster‐based statistics for the KaiC‐KaiB complex. HADDOCK score single terms are averaged over the top 4 members of each cluster. Clusters are ordered according to the averaged HADDOCK score (a.u.). Evdw: Lennard‐Jones potential. Eelec: Coulomb potential. EAIR: Ambiguous interaction restraints energy. Edesolv: Empirical desolvation score. BSA: Buried surface area. The CAPRI docking quality metrics are as follows: interface RMSD (i‐RMSD), fraction of native contacts (FNAT), ligand RMSD (l‐RMSD) and DOCKQ.
**Table SI‐6**: Cluster‐based statistics for the CG workflow of the PRC1 complex. HADDOCK score and its components are reported as averages over the top 4 members of each cluster. The clusters are ordered according to the averaged HADDOCK score (a.u.). Evdw: Lennard‐Jones potential. Eelec: Coulomb potential. EAIR: Ambiguous interaction restraints energy. Edesolv: Empirical desolvation score. BSA: Buried surface area. The CAPRI docking quality metrics are as follows: interface RMSD (i‐RMSD), fraction of native contacts (FNAT), ligand RMSD (l‐RMSD) and DOCKQ.

## Data Availability

The protein–protein and protein‐DNA benchmarking data (in HADDOCK‐ready format) are available from https://github.com/haddocking/BM5-clean and https://github.com/haddocking/Prot-DNABenchmark respectively. All input data and results for the three large complexes used to illustrate the coarse‐grained protocols are available from Zenodo (DOI: 10.5281/zenodo.19551728). The HADDOCK3 software can be freely obtained from https://github.com/haddocking/haddock3.

## References

[pro70793-bib-0001] Abramson J , Adler J , Dunger J , Evans R , Green T , Pritzel A , et al. Accurate structure prediction of biomolecular interactions with AlphaFold 3. Nature. 2024;630:493–500. 10.1038/s41586-024-07487-w PMC11168924 38718835

[pro70793-bib-0002] Ambrosetti F , Jandova Z , Bonvin AMJJ . Information‐driven antibody–antigen modelling with HADDOCK. In: Tsumoto K , Kuroda D , editors. Computer‐aided antibody design. New York, NY: Springer US; 2023. p. 267–282. 10.1007/978-1-0716-2609-2_14 36346597

[pro70793-bib-0003] Baek M , DiMaio F , Anishchenko I , Dauparas J , Ovchinnikov S , Lee GR , et al. Accurate prediction of protein structures and interactions using a three‐track neural network. Science. 2021;373:871–876. 10.1126/science.abj8754 PMC7612213 34282049

[pro70793-bib-0004] Baek M , McHugh R , Anishchenko I , Jiang H , Baker D , DiMaio F . Accurate prediction of protein‐nucleic acid complexes using RoseTTAFoldNA. Nat Methods. 2024;21:117–121. 10.1038/s41592-023-02086-5 PMC10776382 37996753

[pro70793-bib-0005] Basu S , Wallner B . DockQ: a quality measure for protein–protein docking models. PLoS One. 2016;11:e0161879. 10.1371/journal.pone.0161879 PMC4999177 27560519

[pro70793-bib-0006] Bentley ML , Corn JE , Dong KC , Phung Q , Cheung TK , Cochran AG . Recognition of UbcH5c and the nucleosome by the Bmi1/Ring1b ubiquitin ligase complex. EMBO J. 2011;30:3285–3297. 10.1038/emboj.2011.243 PMC3160663 21772249

[pro70793-bib-0007] Brünger AT , Adams PD , Clore GM , DeLano WL , Gros P , Grosse‐Kunstleve RW , et al. Crystallography & NMR system: a new software suite for macromolecular structure determination. Acta Crystallogr Sect D. 1998;54:905–921. 10.1107/s0907444998003254 9757107

[pro70793-bib-0044] ByteDance AML AI4Science Team, Chen X , Zhang Y, Lu C, Ma W, et al. Protenix—advancing structure prediction through a comprehensive AlphaFold3 reproduction. 2025.01.08.631967 10.1101/2025.01.08.631967

[pro70793-bib-0008] de Vries S , Zacharias M . Flexible docking and refinement with a coarse‐grained protein model using ATTRACT. Proteins. 2013;81:2167–2174. 10.1002/prot.24400 23996217

[pro70793-bib-0009] de Vries SJ , van Dijk ADJ, Krzeminski M , et al. HADDOCK versus HADDOCK: new features and performance of HADDOCK2.0 on the CAPRI targets. Proteins Struct Funct Bioinform. 2007;69:726–733. 10.1002/prot.21723 17803234

[pro70793-bib-0010] Evans R , O’Neill M , Pritzel A , et al. Protein complex prediction with AlphaFold‐multime. 2021 10.1101/2021.10.04.463034

[pro70793-bib-0011] Fernández‐Recio J , Totrov M , Abagyan R . Identification of protein–protein interaction sites from docking energy landscapes. J Mol Biol. 2004;335:843–865. 10.1016/j.jmb.2003.10.069 14687579

[pro70793-bib-0012] Giulini M , Reys V , Teixeira JMC , Jiménez‐García B , V. Honorato R , Kravchenko A , et al. HADDOCK3: a modular and versatile platform for integrative modeling of biomolecular complexes. J Chem Inf Model. 2025;65:7315–7324. 10.1021/acs.jcim.5c00969 PMC12264957 40526044

[pro70793-bib-0013] Giulini M , Schneider C , Cutting D , Desai N , Deane CM , Bonvin AMJJ . Towards the accurate modelling of antibody−antigen complexes from sequence using machine learning and information‐driven docking. Bioinformatics. 2024;40:btae583. 10.1093/bioinformatics/btae583 PMC11483107 39348157

[pro70793-bib-0014] Hayashi F , Iwase R , Uzumaki T , Ishiura M . Hexamerization by the N‐terminal domain and intersubunit phosphorylation by the C‐terminal domain of cyanobacterial circadian clock protein KaiC. Biochem Biophys Res Commun. 2006;348:864–872. 10.1016/j.bbrc.2006.07.143 16901465

[pro70793-bib-0015] Hayashi F , Suzuki H , Iwase R , Uzumaki T , Miyake A , Shen JR , et al. ATP‐induced hexameric ring structure of the cyanobacterial circadian clock protein KaiC. Genes Cells. 2003;8:287–296. 10.1046/j.1365-2443.2003.00633.x 12622725

[pro70793-bib-0016] Honorato RV , Roel‐Touris J , Bonvin AMJJ . MARTINI‐based protein‐DNA coarse‐grained HADDOCKing. Front Mol Biosci. 2019;6:102. 10.3389/fmolb.2019.00102 PMC6779769 31632986

[pro70793-bib-0017] Honorato RV , Trellet ME , Jiménez‐García B , Schaarschmidt JJ , Giulini M , Reys V , et al. The HADDOCK2.4 web server for integrative modeling of biomolecular complexes. Nat Protoc. 2024;19:3219–3241. 10.1038/s41596-024-01011-0 38886530

[pro70793-bib-0018] Ishiura M , Kutsuna S , Aoki S , Iwasaki H , Andersson CR , Tanabe A , et al. Expression of a gene cluster kaiABC as a circadian feedback process in cyanobacteria. Science. 1998;281:1519–1523. 10.1126/science.281.5382.1519 9727980

[pro70793-bib-0019] Jorgensen WL , Tirado‐Rives J . The OPLS [optimized potentials for liquid simulations] potential functions for proteins, energy minimizations for crystals of cyclic peptides and crambin. J Am Chem Soc. 1988;110:1657–1666. 10.1021/ja00214a001 27557051

[pro70793-bib-0020] Jumper J , Evans R , Pritzel A , Green T , Figurnov M , Ronneberger O , et al. Highly accurate protein structure prediction with AlphaFold. Nature. 2021;596:583–589. 10.1038/s41586-021-03819-2 PMC8371605 34265844

[pro70793-bib-0021] Karaca E , Bonvin AMJJ . Advances in integrative modeling of biomolecular complexes. Methods San Diego Calif. 2013;59:372–381. 10.1016/j.ymeth.2012.12.004 23267861

[pro70793-bib-0022] Krishna R , Wang J , Ahern W , Sturmfels P , Venkatesh P , Kalvet I , et al. Generalized biomolecular modeling and design with RoseTTAFold all‐atom. Science. 2024;384:eadl2528.10.1126/science.adl252838452047

[pro70793-bib-0023] Kurcinski M , Jamroz M , Blaszczyk M , Kolinski A , Kmiecik S . CABS‐dock web server for the flexible docking of peptides to proteins without prior knowledge of the binding site. Nucleic Acids Res. 2015;43:W419–W424. 10.1093/nar/gkv456 PMC4489223 25943545

[pro70793-bib-0024] Kynast P , Derreumaux P , Strodel B . Evaluation of the coarse‐grained OPEP force field for protein‐protein docking. BMC Biophys. 2016;9:4. 10.1186/s13628-016-0029-y PMC4839147 27103992

[pro70793-bib-0025] Lee B , Richards FM . The interpretation of protein structures: estimation of static accessibility. J Mol Biol. 1971;55:379‐IN4. 10.1016/0022-2836(71)90324-x 5551392

[pro70793-bib-0026] Lensink MF , Wodak SJ . Docking and scoring protein interactions: CAPRI 2009. Proteins. 2010;78:3073–3084. 10.1002/prot.22818 20806235

[pro70793-bib-0027] Lin Z , Akin H , Rao R , Hie B , Zhu Z , Lu W , et al. Evolutionary‐scale prediction of atomic‐level protein structure with a language model. Science. 2023;379:1123–1130. 10.1126/science.ade2574 36927031

[pro70793-bib-0028] Lynch EM , Hansen H , Salay L , Cooper M , Timr S , Kollman JM , et al. Structural basis for allosteric regulation of human phosphofructokinase‐1. Nat Commun. 2024;15:7323. 10.1038/s41467-024-51808-6 PMC11345425 39183237

[pro70793-bib-0029] Marrink SJ , Risselada HJ , Yefimov S , Tieleman DP , de Vries AH . The MARTINI force field: coarse grained model for biomolecular simulations. J Phys Chem B. 2007;111:7812–7824. 10.1021/jp071097f 17569554

[pro70793-bib-0030] Mattiroli F , Uckelmann M , Sahtoe DD , van Dijk WJ , Sixma TK . The nucleosome acidic patch plays a critical role in RNF168‐dependent ubiquitination of histone H2A. Nat Commun. 2014;5:3291. 10.1038/ncomms4291 PMC3929782 24518117

[pro70793-bib-0031] McGinty RK , Henrici RC , Tan S . Crystal structure of the PRC1 ubiquitylation module bound to the nucleosome. Nature. 2014;514:591–596. 10.1038/nature13890 PMC4215650 25355358

[pro70793-bib-0032] Monticelli L , Kandasamy SK , Periole X , Larson RG , Tieleman DP , Marrink SJ . The MARTINI coarse‐grained force field: extension to proteins. J Chem Theory Comput. 2008;4:819–834. 10.1021/ct700324x 26621095

[pro70793-bib-0033] Nooren IMA , Thornton JM . Structural characterisation and functional significance of transient protein–protein interactions. J Mol Biol. 2003;325:991–1018. 10.1016/s0022-2836(02)01281-0 12527304

[pro70793-bib-0034] O'Neill JS , Reddy AB . Circadian clocks in human red blood cells. Nature. 2011;469:498–503. 10.1038/nature09702 PMC3040566 21270888

[pro70793-bib-0035] Pattanayek R , Egli M . Crystal Structure of Full Length Circadian Clock Protein KaiC with Correct Geometry at Phosphorylation Sites: 3dvl. 2009 10.2210/pdb3dvl/pdb

[pro70793-bib-0036] Rader A . Coarse‐grained models: getting more with less. Curr Opin Pharmacol. 2010;10:753–759. 10.1016/j.coph.2010.09.003 20888293

[pro70793-bib-0037] Ranaudo A , Giulini M , Pelissou Ayuso A , Bonvin AMJJ . Modeling protein–glycan interactions with HADDOCK. J Chem Inf Model. 2024;64:7816–7825. 10.1021/acs.jcim.4c01372 PMC11480977 39360946

[pro70793-bib-0038] Rodrigues JPGLM , Bonvin AMJJ . Integrative computational modeling of protein interactions. FEBS J. 2014;281:1988–2003. 10.1111/febs.12771 24588898

[pro70793-bib-0039] Roel‐Touris J , Don CGV , Honorato R , Rodrigues JPGLM , Bonvin AMJJ . Less is more: coarse‐grained integrative modeling of large biomolecular assemblies with HADDOCK. J Chem Theory Comput. 2019;15:6358–6367. 10.1021/acs.jctc.9b00310 PMC6854652 31539250

[pro70793-bib-0040] Saunders MG , Voth GA . Coarse‐graining of multiprotein assemblies. Curr Opin Struct Biol. 2012;22:144–150. 10.1016/j.sbi.2012.01.003 22277168

[pro70793-bib-0041] Snijder J , Burnley RJ , Wiegard A , Melquiond ASJ , Bonvin AMJJ , Axmann IM , et al. Insight into cyanobacterial circadian timing from structural details of the KaiB–KaiC interaction. Proc Natl Acad Sci. 2014;111:1379–1384. 10.1073/pnas.1314326111 PMC3910634 24474762

[pro70793-bib-0042] Snijder J , Schuller JM , Wiegard A , Lössl P , Schmelling N , Axmann IM , et al. Structures of the cyanobacterial circadian oscillator frozen in a fully assembled state. Science. 2017;355:1181–1184. 10.1126/science.aag3218 28302852

[pro70793-bib-0043] Takada S . Coarse‐grained molecular simulations of large biomolecules. Curr Opin Struct Biol. 2012;22:130–137. 10.1016/j.sbi.2012.01.010 22365574

[pro70793-bib-0045] Tseng R , Goularte NF , Chavan A , Luu J , Cohen SE , Chang YG , et al. Structural basis of the day‐night transition in a bacterial circadian clock. Science. 2017;355:1174–1180. 10.1126/science.aag2516 PMC5441561 28302851

[pro70793-bib-0046] Uusitalo JJ , Ingólfsson HI , Akhshi P , Tieleman DP , Marrink SJ . Martini coarse‐grained force field: extension to DNA. J Chem Theory Comput. 2015;11:3932–3945. 10.1021/acs.jctc.5b00286 26574472

[pro70793-bib-0047] van Dijk M , Bonvin AMJJ . A protein–DNA docking benchmark. Nucleic Acids Res. 2008;36:e88. 10.1093/nar/gkn386 PMC2504314 18583363

[pro70793-bib-0048] van Dijk M , Bonvin AMJJ . Pushing the limits of what is achievable in protein‐DNA docking: benchmarking HADDOCK's performance. Nucleic Acids Res. 2010;38:5634–5647. 10.1093/nar/gkq222 PMC2943626 20466807

[pro70793-bib-0049] Villarreal SA , Pattanayek R , Williams DR , Mori T , Qin X , Johnson CH , et al. CryoEM and molecular dynamics of the circadian KaiB–KaiC complex indicates that KaiB monomers interact with KaiC and block ATP binding clefts. J Mol Biol. 2013;425:3311–3324. 10.1016/j.jmb.2013.06.018 PMC3940072 23796516

[pro70793-bib-0050] Vreven T , Moal IH , Vangone A , Pierce BG , Kastritis PL , Torchala M , et al. Updates to the integrated protein‐protein interaction benchmarks: docking benchmark version 5 and affinity benchmark version 2. J Mol Biol. 2015;427:3031–3041. 10.1016/j.jmb.2015.07.016 PMC4677049 26231283

[pro70793-bib-0051] Wass MN , David A , Sternberg MJ . Challenges for the prediction of macromolecular interactions. Curr Opin Struct Biol. 2011;21:382–390. 10.1016/j.sbi.2011.03.013 21497504

[pro70793-bib-0052] Xu X , Giulini M , Bonvin AMJJ . Improved prediction of antibody and their complexes with clustered generative modelling ensembles. Bioinformatics Adv. 2025;5:vbaf161. 10.1093/bioadv/vbaf161 PMC12279294 40692622

